# Association between chronic stress-induced structural abnormalities in Ranvier nodes and reduced oligodendrocyte activity in major depression

**DOI:** 10.1038/srep23084

**Published:** 2016-03-15

**Authors:** Shingo Miyata, Manabu Taniguchi, Yoshihisa Koyama, Shoko Shimizu, Takashi Tanaka, Fumihiko Yasuno, Akihide Yamamoto, Hidehiro Iida, Takashi Kudo, Taiichi Katayama, Masaya Tohyama

**Affiliations:** 1 Division of Molecular Brain Science, Research Institute of Traditional Asian Medicine, Kinki University, Osaka-sayama, Osaka 589-8511, Japan; 2 Department of Anatomy and Neuroscience, Graduate School of Medicine, Osaka University, Suita, Osaka 565-0871, Japan; 3 Department of Psychiatry, Nara Medical University, Kashihara, Nara 634-8522, Japan; 4 Department of Investigative Radiology, National Cerebral and Cardiovascular Center, Suita, Osaka 565-8565, Japan; 5 Department of Psychiatry, Osaka University Health Care Center, Toyonaka, Osaka 560-0043, Japan; 6 Department of Psychiatry, Osaka University Graduate School of Medicine, Suita, Osaka 565-0871, Japan; 7 Department of Child Development and Molecular Brain Science, United Graduate School of Child Development, Osaka University, Kanazawa University and Hamamatsu University School of Medicine, Suita, Osaka 565-0871, Japan; 8 Osaka Prefectural Hospital Organization, Osaka 558-8558, Japan

## Abstract

Repeated stressful events are associated with the onset of major depressive disorder (MDD). We previously showed oligodendrocyte (OL)-specific activation of the serum/glucocorticoid-regulated kinase (SGK)1 cascade, increased expression of axon-myelin adhesion molecules, and elaboration of the oligodendrocytic arbor in the corpus callosum of chronically stressed mice. In the current study, we demonstrate that the nodes and paranodes of Ranvier in the corpus callosum were narrower in these mice. Chronic stress also led to diffuse redistribution of Caspr and Kv 1.1 and decreased the activity in white matter, suggesting a link between morphological changes in OLs and inhibition of axonal activity. OL primary cultures subjected to chronic stress resulted in SGK1 activation and translocation to the nucleus, where it inhibited the transcription of *metabotropic glutamate receptors* (*mGluRs*). Furthermore, the cAMP level and membrane potential of OLs were reduced by chronic stress exposure. We showed by diffusion tensor imaging that the corpus callosum of patients with MDD exhibited reduced fractional anisotropy, reflecting compromised white matter integrity possibly caused by axonal damage. Our findings suggest that chronic stress disrupts the organization of the nodes of Ranvier by suppressing mGluR activation in OLs, and that specific white matter abnormalities are closely associated with MDD onset.

Major depressive disorder (MDD) is a multifactorial disease arising from both environmental and genetic factors; however, the responsible genes and mechanisms of pathogenesis are not fully known. Among many potential environmental factors, repeated stressful events have been linked to the onset of MDD^1^. Recently, brain imaging and postmortem evaluations of human brain tissue revealed that patients with MDD have white matter or oligodendrocyte (OL) abnormalities^2^. In addition, rodent models of stress have found decreased numbers of cortical and amygdalar OLs and neural/glial antigen (NG)2-positive OL precursor cells (OPCs) in these individuals^3^,^4^, suggesting potential links between disturbed myelination and mood disorders.

We previously reported that chronically stressed mice with elevated plasma levels of corticosterone exhibit increased serum/glucocorticoid-inducible kinase (SGK)1 phosphorylation in OLs located in nerve fiber bundles such as the corpus callosum^5^,^6^,^7^. We also found that the expression of adhesion molecules such as N-cadherin and α- and β-catenin was upregulated and that OL morphology was altered by chronic stress^5^,^6^,^7^. Conduction velocity has been shown to be affected by glutamate-induced modulation of mature OL activity^8^, and that the resting membrane potential of OLs is regulated by cyclic (c)AMP level^9^. These findings suggest that SGK1 phosphorylation is associated with the regulation of OL metabotropic glutamate receptor (mGluR) activity, which is known to regulate cAMP levels.

In order to clarify the relationship between excessive arborization of oligodendrocytic processes in rodent models of MDD and the molecular pathogenesis of this disorder in humans, in this study, we investigated the mechanisms underlying myelin-axon interactions at nodes and paranodes of the corpus callosum. To this end, we analyzed the expression of several adhesion molecules and channels identified in the nodes of Ranvier in mice by immunohistochemistry^10^,^11^,^12^. We also examined the corpus callosum of MDD patients by diffusion tensor imaging (DTI) to determine whether white matter abnormalities similar to those in mice exist in humans. Our results suggest that axonal damage due to dysregulation of OLs and nodes of Ranvier is closely associated with the development of MDD.

## Results

### Chronic stress disrupts the morphology and region-specific distribution of proteins at the nodes and paranodes of Ranvier in the corpus callosum

**Figure 1:**
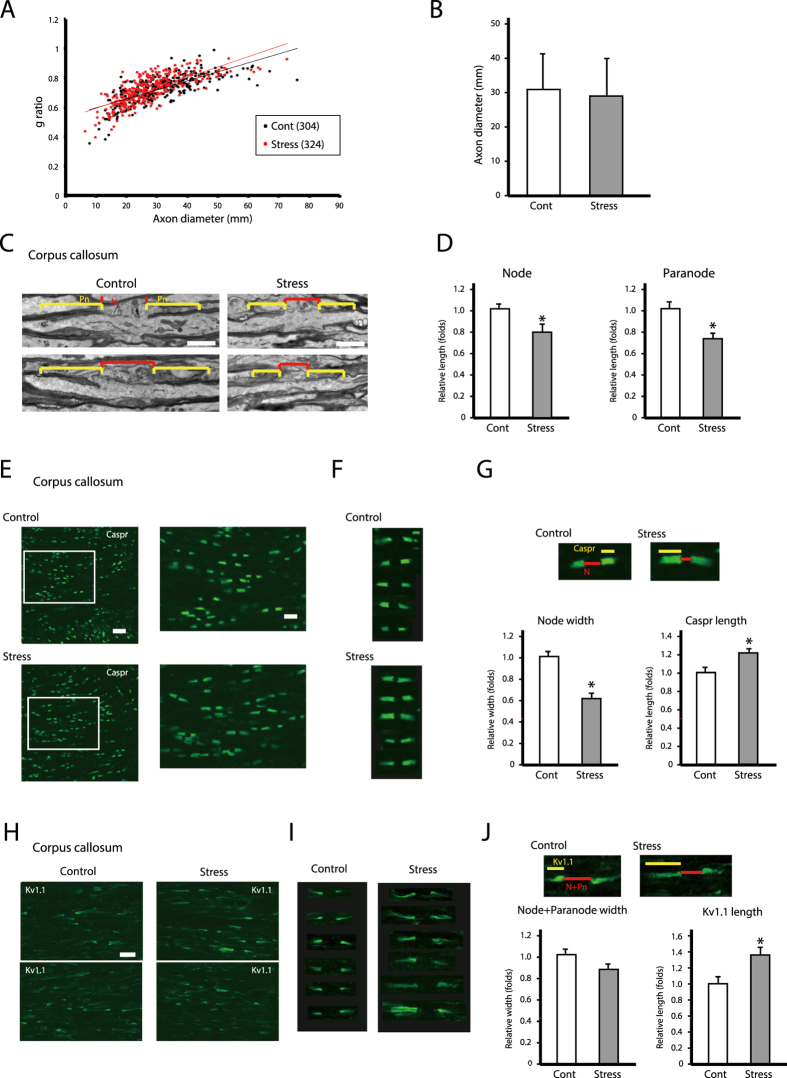
Chronic stress exposure causes morphological alterations in nodes and paranodes of Ranvier of the corpus callosum. (**A**) Axon size and myelin thickness measured by g ratio (the ratio of the inner axonal diameter to the total outer diameter). The distribution of the g ratio is plotted as a function of axonal diameter. The results are expressed as the mean of six images (Cont; 304 axons, Stress; 324 axon) obtained from three independent experiments. *P < 0.05, Student’s *t* test. Cont; non-stressed control mice data, Stress; chronic stress-exposed mice data. (**B**) The distribution of the axon diameters of the corpus callosum in the control and repeated WIRS-exposed mice was assessed. Results are expressed as the mean of six images (300 nodes of Ranvier) obtained from three independent experiments. The results are expressed as the mean ± SEM. *P < 0.05, Student’s *t* test. (**C**) Electron micrographs of nodes (N) and paranodes (Pn) of Ranvier in longitudinal sections of the corpus callosum from control (left panels) and chronically stressed (right panels) mice. Scale bars, 1 μm. (**D**) Measurement of node width and paranode length. Results are expressed as mean ± SEM of six measurements from three independent experiments. *P < 0.05, Student’s *t* test. (**E**,**H**) Immunohistochemical analyses of Caspr (**E**) and K _v_1.1 (**H**) expression in longitudinal sections of the corpus callosum from control (upper panels) and chronically stressed (lower panels) mice. Scale bar, 20 μm. (**F**,**I**) Representative images of Caspr (**F**) and K _v_1.1 (I) expression. Scale bar, 5 μm. (**G**,**J**) Measurement of node width and the length of Caspr-labeled regions at paranodes (**G**), and node and paranode width and length of K _v_1.1-labeled regions at juxtaparanodes (**J**). Results are expressed as the mean of six images (300 nodes of Ranvier) obtained from three independent experiments. Cas, Caspr-labeled region; N, node region; K _v_, K _v_1.1-labeled region; N + Pn, node and paranode region. *P < 0.05, Student’s *t* test.

We previously reported that in the corpus callosum of mice exposed to chronic stress, oligodendrocytic processes are thicker, more numerous, and longer^5^,^6^,^7^. We hypothesized that these morphological changes affect the morphology and function of nodes of Ranvier and axonal projections. Firstly, to confirm the validity of our brain samples for structural analysis, we conducted g ratio analyses and measured the diameter of nerve fibers in the corpus callosum. There were no significant changes in the g ratio or average diameter of the nerve fibers in the corpus callosum of stress-exposed mice relative to the controls (Fig. 1A,B). We therefore carried out comparative ultrastructural analyses of the nodes of Ranvier at the same brain levels in chronically stressed and control mice. Chronic stress reduced the lengths of axon nodes and paranodes in the corpus callosum by approximately 20% relative to those in control mice (Fig. 1C,D). We did not find any additional lesions such as hypo- or demyelination in the corpus callosum of either group (Fig. 1A–D). These results indicated that decreases of nodal and paranodal lengths in the corpus callosum might be induced by chronic stress exposure.

Previous studies have reported several region-specific adhesion molecules and channels at the nodes of Ranvier^10^,^11^,^12^. To investigate their roles in the morphological changes induced by chronic stress exposure, we examined the expression of contactin-associated protein (Caspr) and voltage-dependent potassium channel (K _v_)1.1 (paranodal and juxtaparanodal protein, respectively) in the corpus callosum.

Areas of Caspr immunoreactivity in the paranodes of Ranvier were smaller in chronically stressed as compared to control mice (Fig. 1E,F). Node width, defined as the length of Caspr-positive structures, was reduced by 55% in stressed as compared to control mice (Fig. 1G, Node width). However, the distribution of the Caspr-positive area was more diffuse in the stress group than in controls (Fig. 1G, Caspr length).

Similar to the distribution of Caspr, areas of K _v_1.1 immunoreactivity in juxtaparanodes of Ranvier were smaller in chronically stressed mice than in control mice (Fig. 1H,I): the nodes and paranodes of Ranvier in the former group were approximately 0.85 times the length of the same regions in the latter group (Fig. 1J; Node + Paranode width). However, the distribution of K _v_1.1—that is, the K _v_1.1 cluster—was significantly more diffuse in stressed as compared to control mice (Fig. 1J; K _v_.1.1 length).

### Caspr and K_v_1.1 expression domains overlap in corpus callosum boundary regions in chronically stressed mice

**Figure 2:**
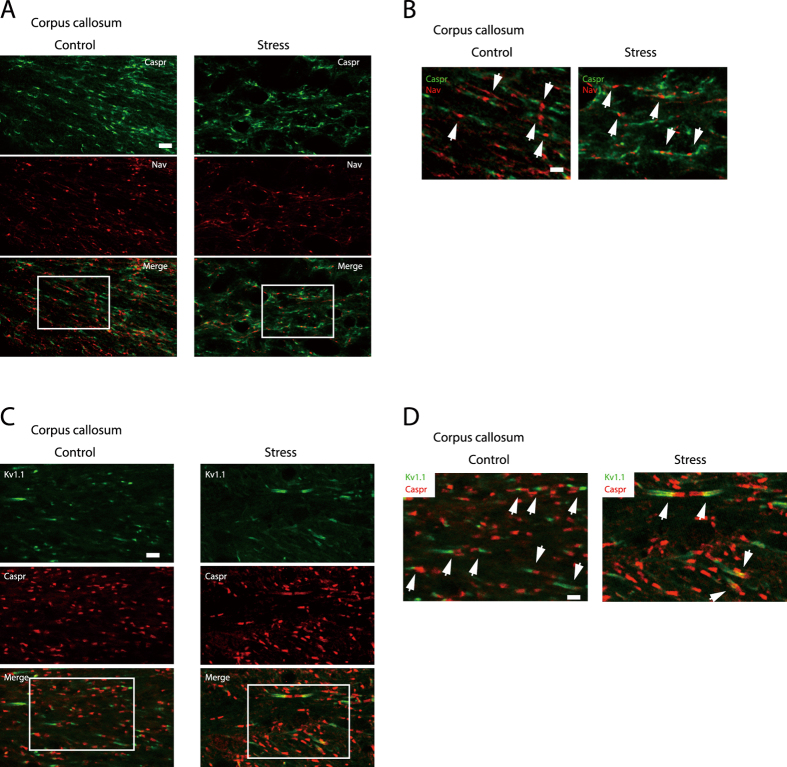
Increase in the length of Caspr- and K _v_1.1-immunoreactive regions at paranodes and juxtaparanodes of the corpus callosum upon chronic stress exposure. (**A**,**C**) Immunohistochemical analyses of Na_v_ and Caspr (**A**), and K _v_1.1 and Caspr (**C**) expression in longitudinal sections of the corpus callosum from control (left panels) and chronically stressed (right panels) mice. Scale bar, 10 μm. (**B**,**D**) Enlargement of the areas enclosed by squares in (**A**,**C**). Scale bar, 5 μm. Arrows indicate Na_v_- and Caspr-positive (**B**) and K _v_1.1- and Caspr-positive (**D**) boundary regions.

To determine whether narrowing of the nodal regions affects Na_v_ localization, we examined voltage-dependent sodium channel (Na_v_) and Caspr expression in the nodal and paranodal regions, respectively, by immunocytochemistry^12^,^13^,^14^. Na_v_ localization was distinct from Caspr clusters in the corpus callosum of both control and chronically stressed mice (Fig. 2A,B).

To clarify the relationship between the narrowing of paranodal regions and the diffuse distributions of Caspr and K _v_1.1, the expression of these two molecules in the paranodal and juxtaparanodal regions was examined by immunocytochemistry^11^,^13^,^15^. In the corpus callosum of control mice, Caspr and K _v_1.1 were expressed in distinct domains (Fig. 2C,D; Control); however, in stressed mice, the distribution of the two proteins overlapped at paranode/juxtaparanode boundaries (Fig. 2C,D; Stress).

### Adhesion molecule expression levels of adjacent nodes of Ranvier are altered after chronic stress exposure

We next investigated the expression levels of adhesion molecules required for axon-myelin interactions^13^,^16^,^17^,^18^,^19^,^20^. We obtained protein samples not only from the corpus callosum, but also from the frontal cortex and hippocampus for western blotting using a brain slicer. The expression levels of 2′,3′-cyclic nucleotide 3′-phosphodiesterase (CNPase) and N-myc downstream regulated (NDRG)1 (myelin and OL markers, respectively) in the corpus callosum were significantly higher than in the frontal cortex and hippocampus (Supplementary Fig. S1A,B). Furthermore, neuronal nuclei (a neural marker) expression was scarcely detected in corpus callosum samples (Supplementary Fig. S1A,B), confirming that they were suitable for examining protein expression levels in specific regions of the corpus callosum.

**Figure 3:**
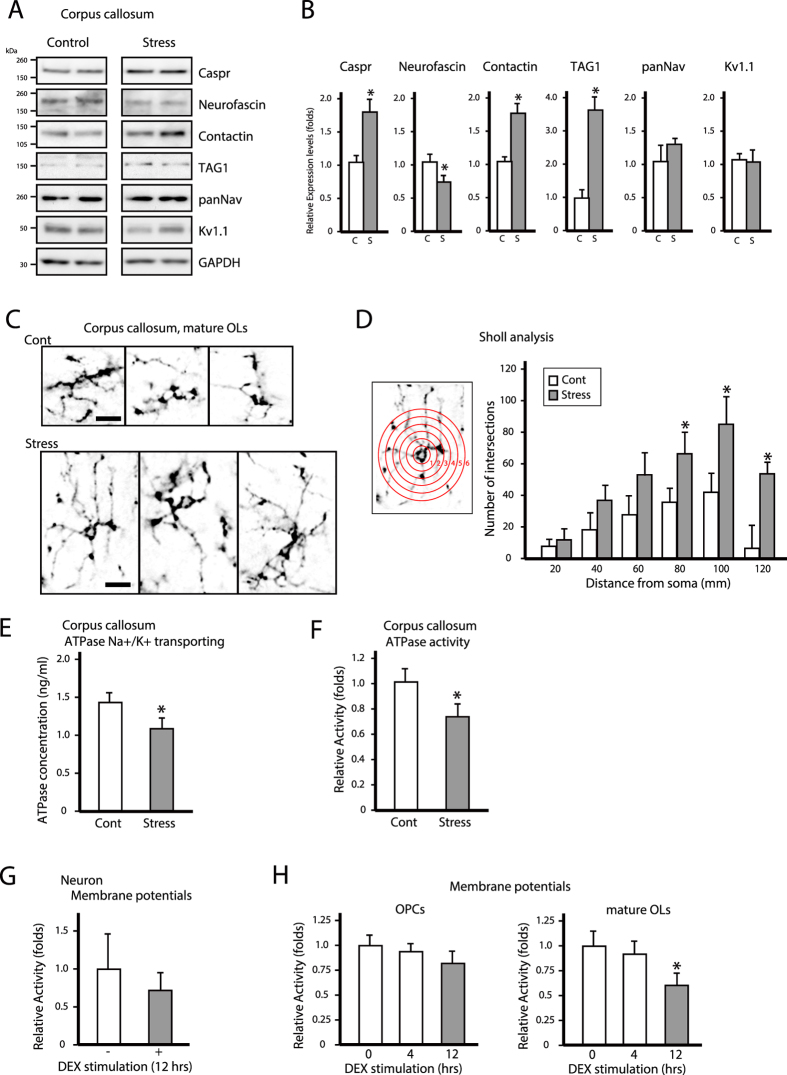
Chronic stress exposure leads to upregulation of adhesion molecules at adjacent nodes of Ranvier, causes morphological changes in mature oligodendrocytes, and decreases axonal activity in the corpus callosum. (**A**) Western blot analysis of the expression of adhesion molecules (Caspr, total neurofascin, contactin, TAG1) and channels (Na_v_ and K _v_1.1) at adjacent nodes of Ranvier in the corpus callosum of control and chronically stressed mice. (**B**) Quantification of protein bands from (**A**). Data are expressed as mean ± SEM of at least three independent experiments. *P < 0.05, Student’s *t* test. (**C**) Representative images of the processes of oligodendrocytes in control (Cont) and chronic stress exposed (Stress) mice. Scale bar, 40 μm. (**F**) Process complexity and branching are indicated by Sholl analysis. Quantification of intersection numbers within each radius (1–6). Results are the mean of six images obtained from three independent experiments and are expressed as the mean ± SEM. *P < 0.05, Student’s *t* test. (**E**,**F**) Na+/K+ transporting ATPase levels (**E**) and Na+/K+-ATPase activity (**F**) in the corpus callosum of control and chronically stressed mice. Data are expressed as mean ± SEM of at least three independent experiments. *P < 0.05, Student’s *t* test. (**G**,**H**) Time course of membrane potential activities of OPCs and mature OLs primary cultures (**G**) and membrane potential activities of 12 h DEX administration primary neurons (**H**) after 100 μM (**G**) or 10 μM (**H**) dexamethasone (DEX) administration. Data are expressed as mean ± SEM of at least three independent experiments. *P < 0.05, Student’s *t* test.

Na_v_ and K _v_1.1 expression was unaltered in the corpus callosum, whereas levels of the paranodal proteins Caspr and contactin as well as the juxtaparanodal protein transient axonal glycoprotein 1 (TAG1) were upregulated and that of total neurofascin (155 and 186 isoforms) was downregulated in response to chronic stress (Fig. 3A,B). These results indicate that chronic stress disrupts normal axon-myelin adhesion.

### Chronic stress induces morphological changes in mature OLs decreases axonal activity of the corpus callosum

We sought to determine whether oligodendrocytic processes induced the axonal branching that was observed following chronic stress exposure. Immature OLs or OPCs were distinguished from mature OLs by the expression of NG2 and adenomatous polyposis coli (APC), respectively^21^,^22^. The morphological complexity of OL processes was quantified by Sholl analysis^23^,^24^. APC-positive OL processes in the corpus callosum of stressed mice were more numerous and thicker than those in control mice (Fig. 3C,D). Furthermore, APC-labeled processes extended longer distances in stressed mice than in controls (Fig. 3C,D and Supplementary Fig. S2A; APC columns). However, chronic stress exposure had no effect on the number or morphology of NG2-labeled OPCs of the corpus callosum (Supplementary Fig. S2A; NG2 columns). These results indicate that chronic stress affects the structure of mature but not immature OLs. Moreover, chronic stress did not lead to microglia activation nor did it increase the number astrocytes in the corpus callosum (Supplementary Fig. S2B).

Since axonal integrity is essential for action potential propagation, any disruption in axon structure is likely to affect its activity. As a measure of axonal integrity, we investigated adenosine triphosphatase (ATPase) concentrations (Na^+^/K^+^ transporting) and ATPase activity in the corpus callosum^25^,^26^,^27^,^28^,^29^. Chronic stress exposure significantly decreased both Na^+^/K^+^-ATPase concentrations and activity in the fiber tract of the corpus callosum (Fig. 3E,F). Furthermore, to investigate the axonal membrane activity, we examined the membrane potentials of the differentiated neuronal cells by using fluorescent dye^30^. The membrane potentials of differentiated neuronal cells decreased after 12 hrs of chronic stress, mimicked by treatment with the synthetic glucocorticoid dexamethasone (DEX) (Fig. 3G). We further examined membrane potentials in the undifferentiated OPC cells and mature OLs. The undifferentiated OPC cells membrane potentials did not show significant change after 12 hrs of DEX stimulation (Fig. 3H). In contrast, membrane potentials of differentiated OLs were significantly decreased after 12 hrs of DEX stimulation (Fig. 3H). These results suggested that chronic stress exposure induced morphological changes in the processes of mature OLs and compromised mature OL membrane function in the corpus callosum. Furthermore, these structural and functional problems in mature OLs might affect axonal activities.

### SGK1 nuclear translocation reduces OL activity by suppressing *mGluR* expression

**Figure 4:**
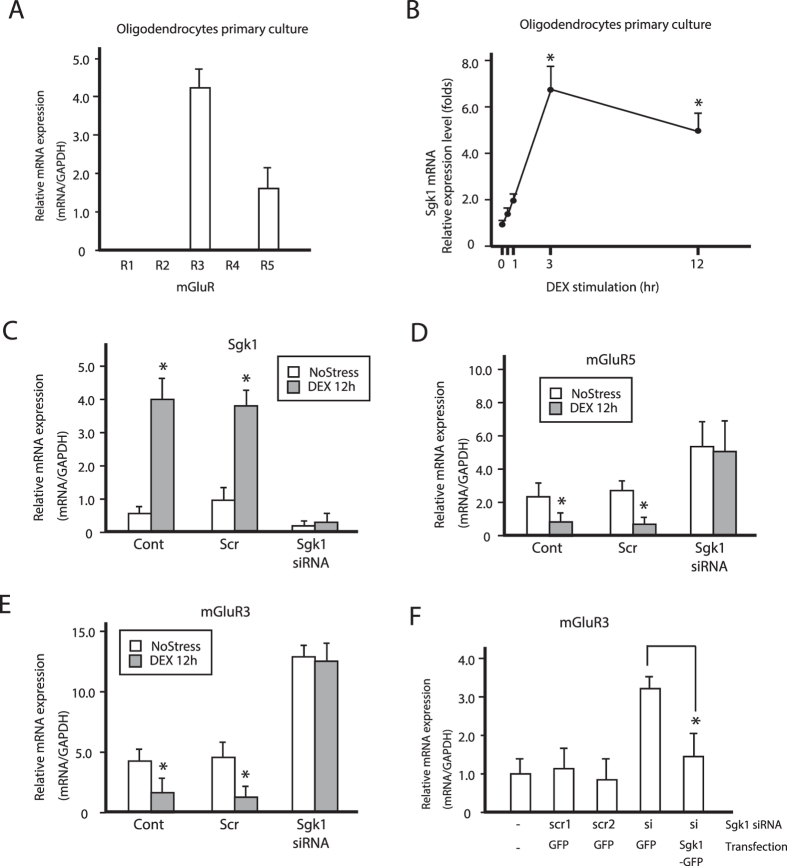
Chronic stress suppresses *mGluR3* and *5* expressions in primary oligodendrocytes. (**A**) *mGluR1* to *5* mRNA expression in oligodendrocytes. Data are expressed as mean ± SEM of at least three independent experiments. *P < 0.05, Student’s *t* test. (**B**) Time course of *Sgk1* mRNA expression in oligodendrocytes after 100 μM DEX administration. Data are expressed as mean ± SEM of at least three independent experiments. *P < 0.05, Student’s *t* test. (**C**–**F**) Real-time PCR analysis of *Sgk1* (**C**), *mGluR5* (**D**), and *mGluR3* (**E**,**F**) mRNA expression in primary oligodendrocytes. Data are expressed as mean ± SEM of at least three independent experiments. *P < 0.05, Student’s *t* test.

**Figure 5:**
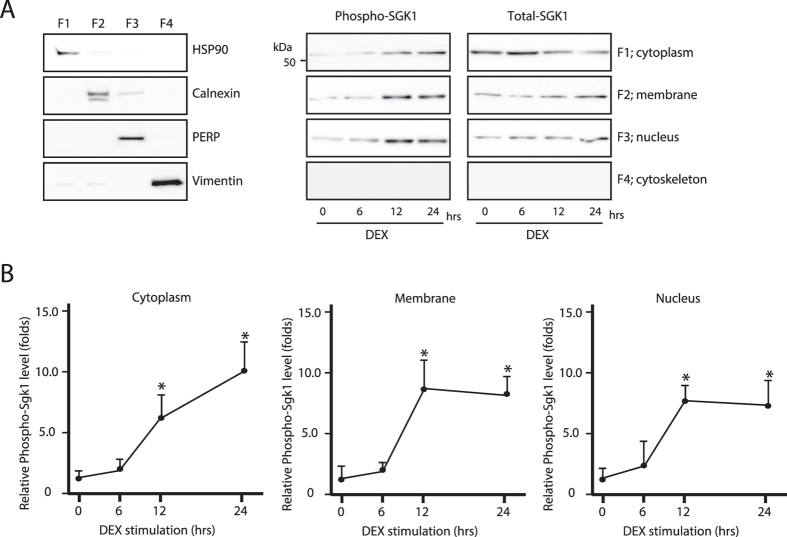
Chronic stress induces nuclear translocation of phospho-SGK1. (**A**) Western blot analysis of total and phosphorylated SGK1 protein levels in cytoplasmic (F1), membrane (F2), nuclear (F3), and cytoskeletal (F4) fractions of primary oligodendrocytes after treatment with 100 μM DEX. (**B**) Quantification of protein bands from (**A**). Data are expressed as mean ± SEM of at least three independent experiments. *P < 0.05, Student’s *t* test.

Glutamate is a major excitatory neurotransmitter in the mammalian central nervous system and modulates mature OL activity^8^,^31^. Previous reports showed that mGluRs are therapeutic targets for the treatment of mood disorders^31^. Several subtypes of mGluRs are associated with adenylate cyclase activity and cAMP levels and are responsible for signal propagation in the central nervous system^31^,^32^,^33^. The regulation of cAMP level is linked to the resting membrane potential of OLs^9^. To determine how morphological changes in OLs modulate neuronal activity, we examined the expression of mGluRs, which are known to regulate cAMP levels in OLs^34^. *mGluR3* and *5* mRNA was expressed in primary cultured OLs (Fig. 4A); transcript levels decreased after chronic DEX administration (Fig. 4D,E). In contrast, *Sgk1* mRNA expression increased after chronic stress exposure (Fig. 4B,C). Chronic stress has been shown to induce the phosphorylation and activation of SGK1 in OLs^5^,^6^,^7^. Upon DEX treatment, SGK1 was phosphorylated and translocated to the nucleus (Fig. 5A,B). Nuclear SGK1 repressed the transcription of *mGluR3* and *5*, since *Sgk1* knockdown resulted in the upregulation of both transcripts (Fig. 4D,E), an effect that was reversed by SGK1 overexpression (Fig. 4F). In addition, the phosphorylation level of NDRG1 in the corpus callosum was increased after chronic stress exposure, while mGluR3 protein expression was downregulated in the corpus callosum (Fig. 4G,H).

**Figure 6:**
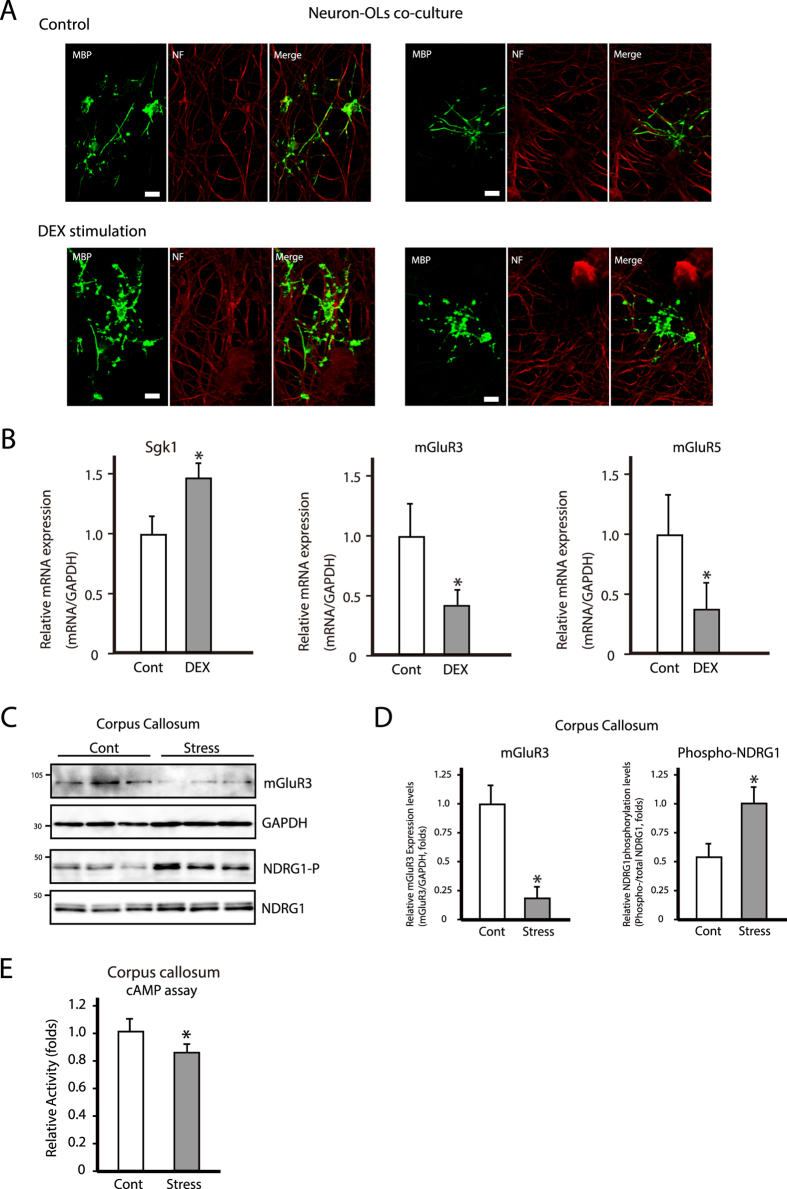
Chronic stress decreases mGluR3 and 5 expressions in a co-culture system and in the corpus callosum, and cAMP level in the corpus callosum. (**A**) Representative images of immunocytochemical analysis of Neurofilament (NF) and MBP in oligodendrocyte and DRG-neuron co-culture system after treatment with (Cont) or without (DEX stimulations) 10 μM DEX for 12 hrs. Scale bar = 20 μm. (**B**) *Sgk1, mGluR3,* and -*5* mRNA expression in co-culture system. Data are expressed as mean ± SEM of at least three independent experiments. *P < 0.05, Student’s *t* test. (**C**) Western blot analysis of mGluR3 and phosphorylated NDRG1 (oligodendrocyte stress marker) in the corpus callosum in control (Cont) and chronic stress-exposed (Stress) mice. (**D**) Quantification of protein bands from (**C**). Data are expressed as mean ± SEM of at least three independent experiments. *P < 0.05, Student’s *t* test. (**E**) cAMP levels in the corpus callosum of control (Cont) and chronically stressed (Stress) mice.

To investigate the relationship between neuronal axons and mature OLs, we established a DRG neuron and OL co-culture system^35^. We investigated mature OL processes and myelin-like sheath formations with and without chronic DEX stimulation. Control mature OLs had several branched processes and myelin-like sheath formations (Fig. 6A; control columns). However, after chronic DEX administration, mature OL processes were more complex and longer than those in control conditions, and the formation levels of myelin-like sheath in chronic stressed conditions decreased in comparison with those in control conditions (Fig. 6A; DEX stimulation columns). We further examined mature OL stress levels by measuring *Sgk1*, *mGluR3,* and -*5* mRNA expression levels in co-culture conditions. Chronic DEX administration increased *Sgk1* mRNA expression in these co-cultures, while both *mGluR3* and -*5* mRNA levels were decreased, as observed in primary OL cultures exposed to chronic stress (Fig. 6B).

We next investigated the relationship between mGluRs protein expression and cAMP levels in the corpus callosum. NDRG1 phosphorylation was increased in the corpus callosum after chronic stress exposure in mice (Fig. 6C,D)^5^ while mGluR3 and cAMP levels were decreased (Fig. 6C–E). These results suggest that chronic stress can suppress OL activity in the corpus callosum by downregulating mGluR expression, and might correspondingly change axon-mature OL interactions.

### MDD patients exhibit axonal white matter abnormalities in the corpus callosum

Our findings in the mouse model indicated that microstructural damage to white matter tracts may predispose humans to depressive symptoms. To investigate this possibility, we used DTI to examine the microstructure of fiber tracts in the corpus callosum of MDD patients^2^,^36^,^37^. DTI combines a conventional magnetic resonance imaging sequence with additional magnetic field gradients to quantify water diffusion by fractional anisotropy (FA), which is a measure of how diffusion is directionally hindered; this allows an assessment of brain white matter integrity.

**Figure 7:**
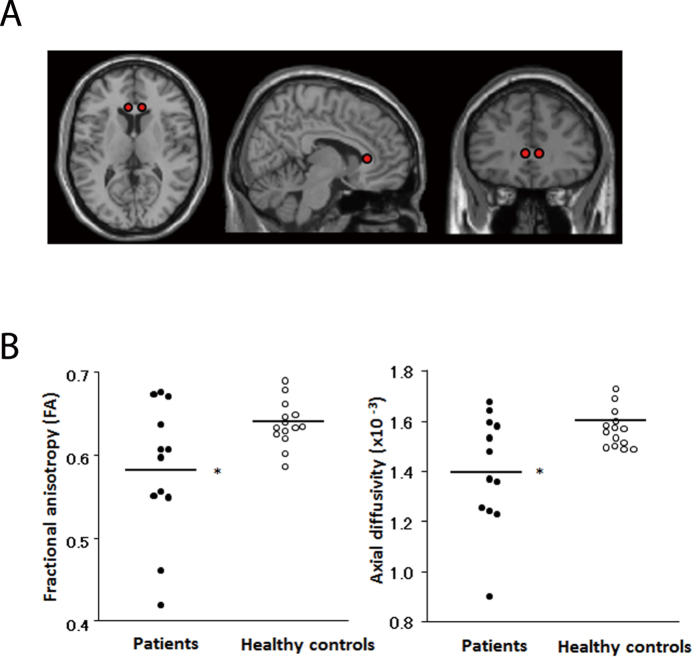
Lower fractional anisotropy (FA) values are observed in patients with MDD compared to healthy control subjects by DTI. (**A**) Spherical voxels of interest (VOIs) placed on the anterior genu of the corpus callosum and (**B**) scatter plots of FA and axial diffusivity values in this region are shown for patients with MDD and controls. *Significantly lower FA and axial diffusivity values were observed in patients than in controls. Values were adjusted to the mean values of age and gender.

In the voxel-based analysis of FA values, there was no difference between patients and healthy controls (P < 0.001, uncorrected). However, by analyzing volumes of interest (VOI) on FA maps of the anterior corpus callosum—where stress-induced abnormalities were observed in mice—we found significantly lower FA values in MDD patients. We also detected reduced axial diffusivity in treatment-naive MDD patients (Fig. 7, Table 1). Taken together, our findings suggest that chronic stress can lead to axonal abnormalities that may underlie the development of MDD.

## Discussion

Many diseases of the nervous system attack non-neuronal cells and indirectly affect the function and integrity of neurons. In the current study, we found that exposure to chronic stress leads to morphological alterations in myelinating OLs of the corpus callosum in mice. Altered oligodendrocytic activity and structure disrupted axon-myelin adhesion at paranodes and nodes of Ranvier, as evidenced by the diffuse distribution of key proteins and reduced axonal activity. Patients with MDD also showed white matter abnormalities in their corpus callosum. We therefore propose that chronic stress undermines axon-myelin interactions and reduces axonal activity, possibly triggering disorders such as MDD.

Neurofascin-deficient mice showed disruption of node and paranode complexes and reduced neural function^38^. Chronic stress induced abnormalities at paranode/juxtaparanode of Ranvier boundary regions in the corpus callosum, although the functional significance of these findings is unclear^39^,^40^. Caspr- and contactin-deficient mice showed junctional disruptions^13^,^16^,^17^,^18^,^19^,^20^. Despite the decrease in the width of nodes of Ranvier in chronically stressed mice, the structure of nodal regions and Na_v_ clusters were preserved by the adherence of paranodal loops^12^,^13^,^14^,^16^,^17^,^18^,^19^,^20^. The upregulation of Caspr and contactin may account for the maintenance of nodal boundaries following chronic stress. The diffuse distribution of Caspr and K _v_1.1 in these boundary regions may alter neuronal activity and axonal conduction velocity, although the latter is reportedly unaffected by diffuse K _v_1.1 distribution at internodes^11^,^15^,^41^. We observed an overlap between Caspr and K _v_1.1 expression domains in boundary regions. Hence, it can be supposed that Caspr upregulation and alterations in the boundary regions modulate axonal conduction velocity or the regulation of neuronal activity by OLs.

Active signal transduction between myelinated axons and the myelin sheath of mature OLs has only recently been recognized^8^,^42^,^43^. Up to 60 axons can be myelinated simultaneously by a single mature OL^44^, which can thus potentially regulate neural transmission in a number of axons within the same fiber assembly. Several recent studies have shown that in animal models of major depression, stressful experiences decreased OL proliferation in the frontal cortex and amygdala^3^,^4^. Interestingly, OLs are reportedly a target of plasma corticosterone^4^,^45^,^46^,^47^. Sgk1 is a glucocorticoid receptor-regulated gene; SGK1 phosphorylation in OLs resulted in its nuclear translocation and negative regulation of mGluR transcription, resulting in decreased cAMP levels in OLs after stress exposure^8^,^9^. Previous reports have suggested mGluR2/3 and 5 as novel therapeutic targets for mood disorders such as MDD and schizophrenia, among others^34^,^48^,^49^,^50^. In various animal models of MDD, mGluR level has been linked to disease pathogenesis and symptoms^51^,^52^; for instance, mGluR deficiency had adverse effects on depressive symptoms^49^,^53^. The results of the present study demonstrate the critical importance of OL mGluR2/3 and 5 function in the treatment of depression, although the detailed molecular mechanisms of how they regulate cAMP levels and thereby contribute to the onset of MDD remain to be elucidated.

Given our observation that axon-myelin structure is disturbed in chronically stressed mice, we investigated whether patients with MDD—a disorder typically arising from repeated bouts of stress—have similarly compromised white matter integrity. As proposed by a previous study^54^, we used DTI to examine microstructural abnormalities in the white matter^2^,^36^,^37^ and found that compared to control subjects, patients with MDD showed reduced FA values in the anterior corpus callosum, as well as a trend of decreased axial but not radial diffusivity. Axonal damage markedly decreases axial diffusivity, while demyelination increases radial diffusivity^55^. Therefore, our findings suggest that chronic stress and MDD do not result in demyelination but rather in alterations to axonal structure, possibly caused by chronic stress-induced dysregulation of nodes of Ranvier and white matter disturbance.

The anterior genu of the corpus callosum is the major commissure connecting homologous regions of the left and right prefrontal, anterior cingulate, and insular cortices. Both the anterior cingulate cortex and insula have connections with limbic structures such as the amygdala, and are involved in affective processing and cognitive functioning^56^. Given that the corpus callosum connects brain regions in the two hemispheres that are important for emotional and cognitive functions, microstructural abnormalities in this region related to the loss of white matter integrity may negatively impact interhemispheric communication and trigger the onset of mood disorders.

In summary, the results of the present study highlight important parallels between abnormalities in fiber projections of patients with MDD and alterations in axonal structure, OL activity, and neural transmission in the chronic stress mouse model of MDD. We demonstrated that Na^+^/K^+^-ATPase activity in axons and cAMP level in OLs were decreased in mice subjected to chronic stress, and that treatment-naive patients with MDD had microstructural abnormalities in fiber projections. We also showed that disturbances in nodes, paranodes, and juxtaparanodes of Ranvier were not linked to demyelinating pathologies. A primary goal of future research will be to clarify the functional implications of these novel findings in the fiber tracts of stressed mice and patients with MDD.

## Methods

### Animals

All animal care and handling procedures were approved by the Institutional Animal Care and Use Committee of Kinki University (no. KAME-24-021). All animal experiments were carried out in accordance with the guidelines set forth in the Guide for the Care and Use of Laboratory Animals (National Institutes of Health, Bethesda, MD, USA).

### Chronic stress exposure

Mice were exposed to chronic stress as previously described^5^ (details are provided in the Supplementary Information).

### Immunohistochemistry

Immunohistochemical analysis was performed as previously described^5^,^57^. Briefly, sections were immersed in pre-warmed 0.1 M citrate buffer (pH 6.0) and boiled for 5 min at 95 °C–100 °C, then allowed to cool for 20 min. The sections were then rinsed with phosphate-buffered saline (PBS) for 30 min and incubated at 4 °C for 24 h with anti-Caspr, anti-K _v_1.1, anti-pan-Na, anti-CD11, anti-GFAP, anti-CC1, and anti-NG2 diluted 1:500 in PBS. The sections were then rinsed with phosphate-buffered saline (PBS) for 30 min and incubated at room temperature for 1 h with Alexa Fluor 488-conjugated goat anti-rabbit or anti-mouse IgG (Life Technologies, Carlsbad, CA, USA) diluted 1:500 in PBS. After a 1-h wash with PBS, sections were mounted on slides using PermaFluor (Thermo Scientific, Waltham, MA, USA) and visualized by confocal microscopy (LSM-510; Carl Zeiss, Oberkochen, Germany) under a 63× objective.

### Electron microscopy

Mice were anesthetized and transcardially perfused with 4% paraformaldehyde and 2% glutaraldehyde (v/v) in 0.1 M PBS. The brain was dissected and dehydrated in a graded series of ethanol (50–100%), then embedded in Epon. Semi-thin sections (0.9 mm) were cut and stained with Toluidine Blue for examination by light microscopy. Ultra-thin sections (80 nm) were cut and stained with 2% uranyl acetate (v/v; Watson’s modified method) and Reynold’s lead citrate, and visualized using a Hitachi H-7650 transmission electron microscope (Tokyo, Japan). Digital images of coronal and longitudinal serial sections were acquired and analyzed using Image Pro Plus 3.0 software (Media Cybernetics, Rockville, MD, USA). The width of nodes and paranodes of Ranvier were counted in 300–1,000 fibers in corpus callosum sections. The g ratio was calculated from the obtained diameters of fibers and axons. Statistical analyses of the measured g ratios were performed using GraphPad Prism 6 (GraphPad, La Jolla, CA, USA).

### DTI study

After providing a complete description of the study, written, informed consent was obtained from each subject. The study was approved by the medical ethics committee of the Osaka University Medical School and the National Cerebral and Cardiovascular Center in Japan, and has been performed in accordance with ethical standards outlined by the Declaration of Helsinki. Additional details are provided in the Supplementary Information.

## Additional Information

**How to cite this article**: Miyata, S. *et al.* Association between chronic stress-induced structural abnormalities in Ranvier nodes and reduced oligodendrocyte activity in major depression. *Sci. Rep.* **6**, 23084; doi: 10.1038/srep23084 (2016).

## Supplementary Material

Supplementary Information

## Figures and Tables

**Table 1:** Differences in values of FA and axial/radial diffusivity in anterior genu of corpus callosum between patients and healthy control subjects.

FA and axial/radial diffusivity	Patients (n = 12)	Healthy controls (n = 14)	Analysis of Covariance^a^	
			F (1, 22)	P
FA	0.58 ± 0.08	0.64 ± 0.03	6.79	0.02 *
Axial diffusivity (×10^−3^)	1.41 ± 0.21	1.57 ± 0.08	5.35	0.007 **
Radial diffusivity (×10^−3^)	0.54 ± 0.06	0.52 ± 0.07	1.16	0.29

Data are mean ± sd. *p < 0.05, **p < 0.01.

^a^Age and gender are entered as covariates.
