# Supplementary material for: Association between chronic stress-induced structural abnormalities in Ranvier nodes and reduced oligodendrocyte activity in major depression
Source: Sci Rep. 2016 Mar 15;6:23084. doi: 10.1038/srep23084 (PMC4791682; doi:10.1038/srep23084)
Supplement: Supplementary Information [file srep23084-s1.pdf]

Supplemental Information

Association between chronic stress-induced structural abnormalities in Ranvier nodes and reduced oligodendrocyte activity in major depression

Shingo Miyata, Manabu Taniguchi, Yoshihisa Koyama, Fumihiko Yasuno, Shoko Shimizu, Takashi Tanaka, Taiichi Katayama, Akihide Yamamoto, Hidehiro Iida, Takashi Kudo, Masaya Tohyama

Supplemental figure legends

Supplementary Figure S1. Quality check of corpus callosum, cortex, and hippocampus tissue samples. (A) Western blot analysis of the myelin (CNPase), oligodendrocytes (NDRG1), and neural (NeuN) markers in the corpus callosum (cc), frontal cortex (Fcx) and hippocampus (Hipp) samples. (B) Quantification of protein bands from (A). Data are expressed as mean ± SEM of at least three independent experiments. *$P < 0.05$, Student's *t* test.

Supplementary Figure S2. Chronic stress exposure causes morphological changes in mature oligodendrocytes in the corpus callosum. (A) Representative images show a greater number of and higher immunoreactivity in the processes of oligodendrocytes in mice exposed to chronic stress as compared to controls. Part of each image is shown at high magnification in the white boxes. Scale bar, 10 μm. (B) Quantitative analysis of NG2 and APC immunoreactivity. The numbers of glial fibrillary acidic protein (GFAP)-positive astrocytes and active, cluster of differentiation (CD)11b-positive microglia were counted. Data are expressed as mean ± SEM of at least three independent experiments. *$P < 0.05$, Student's t test.

Supplemental Materials and Methods

Animals. Adult male C57/BL6 mice weighing 25–35 g were obtained at 11 weeks of age from Japan SLC, Inc. (Hamamatsu, Japan). Three mice per cage were housed in a temperature- (22°C ± 2°C), humidity- (55% ± 10%), and light- (12:12-h light/dark schedule; lights on at 07:00) controlled environment and had free access to laboratory food and water. Mice were allowed to adapt to the experimental environment for 1 week before experiments were performed.

Chronic stress exposure. Mice were placed in a 50-ml conical polypropylene centrifuge tube and immersed vertically to the level of the xiphoid process in a water bath at 23°C for 2 h once daily for 3 weeks. In our preliminary experiments, gastric ulcers were not induced by chronic stress exposure. Control mice were transferred from their home cages to new breeding cages for 2 h. Animals were assigned to experimental groups by means of a completely randomized design. Depression-like behavior was measured with tail suspension and forced swimming tests. The duration of immobility was recorded in both tests during the last 6 min of the 10-min testing period.

Immediately after testing, mice were anesthetized with sodium pentobarbital (30 mg/kg) and perfused transcardially with 4% paraformaldehyde in 0.1 M phosphate buffer. Given the cumulative non-acute effects of stress, to eliminate the influence of the last acute stress exposure and thus evaluate the effects of chronic stress, mice were sacrificed 1 day after the last stress session.

Antibodies. Antibodies against the following proteins were used in this study: contactin-associated protein (Caspr), $K_v1.1$, contactin, and TAG1 (all polyclonal from Abcam, Inc., Cambridge, MA, USA); pan-Na_v (K58/35), Neurofilament 200 (NE14) and CNPase (11-5B) (monoclonal from Sigma Chemical Co., St. Louis, MO, USA); neurofascin (polyclonal), glyceraldehyde-3-phosphate dehydrogenase (GAPDH) (polyclonal) and NDRG1 (polyclonal) and cluster of differentiation (CD)11b (monoclonal) (all from Santa Cruz Biotechnology, Santa Cruz, CA, USA); mGluR3 (polyclonal from LifeSpan BioScience Inc., Seattle, WA, USA); adenomatous polyposis coli (Ab-7) (CC-1) (monoclonal), NeuN (A6) (monoclonal), myelin basic protein (monoclonal), NG2 (132.39) (monoclonal) and glial fibrillary acidic protein

(polyclonal) (all from Millipore, Billerica, MA, USA); Phospho-NDRG1 (Thr346) (D98G11) (polyclonal from Cell Signaling Technology Inc., Danvers, MA, USA).

Preparation corpus callosum tissue samples. Mice were sacrificed and their brains were sliced into coronal or sagittal sections using a Brain Slicer Matrix (slice pitch 1.0 mm each). Sections were immediately placed in ice-cold phosphate-buffered saline (PBS). Gray matter regions around the corpus callosum were carefully removed using a Feather Surgical Blade (no. 11) under a microscope, and corpus callosum tissue was collected in clean tubes.

Sholl analysis. Mature OL morphology was analyzed using the Ghosh lab Sholl Analysis plugin for ImageJ (http://labs.biology.ucsd.edu/ghosh/software/, version 1.0). The program superimposes a grid of concentric circles with increasing radii on an OL cell body, and then measures and combines the number of intersections made by the branching OL processes within each circle (starting radius 10 μm, end: 600 μm, thickness: 100 μm)[1].

Western blot analysis. Western blot analysis was performed as previously described[2,3]. Cells and tissues were washed twice in PBS, harvested, and lysed in TNE buffer composed of 10 mM Tris-HCl (pH 7.8), 1 mM EDTA, and 150 mM NaCl containing 1% (v/v) Nonidet P-40 and a protease inhibitor cocktail (Roche Diagnostics Corp., Indianapolis, IN, USA). Equal amounts of protein were subjected to 7% (v/v) sodium dodecyl sulfate polyacrylamide gel electrophoresis and then transferred to a polyvinylidene difluoride membrane (Millipore). The membrane was blocked with 5% (w/v) skim milk and incubated with primary antibodies followed by incubation with horseradish peroxidase-conjugated secondary antibody. Proteins were visualized with an enhanced chemiluminescence detection system (BD Biosciences, Franklin Lakes, NJ, USA).

Plasmid construction. Green fluorescent protein (GFP)-fused serum/glucocorticoid-regulated kinase (SGK1) under control of the elongation factor (EF)-1α promoter was generated using the pENTR/D-TOPO vector (Life Technologies,

Inc., Carlsbad, CA, USA). The EF-1α promoter was amplified from the pEF5/FRT/V5-D-TOPO vector using the following primer pair: 5'-GCC GCC CCC TTC ACC GGA GTG CCT CGT GAG GCT CCG GTG-3' (forward) and 5'-GGC GCG CCC ACC CTT AGT ACT TCA CGA CAC CTG AAA TGG AAG AAA-3' (reverse). The GFP moiety was amplified from the ppcDNA6.2-GW/EmGFP-miR vector (Life Technologies, Inc.) using the following primer pair: 5'-CAG GTG TCG TGA AGT GCC ACC ATG GTG AGC AAG GGC GAG GAG CTG-3' (forward) and 5'-GCG CCC ACC CTT AGT CTC TAG ATC AAC CAC TTT GTA CAA-3' (reverse). Wild-type mouse *Sgk1* (ΔN-SGK1) was cloned using a PCR-based method. *Sgk1* was amplified with PrimeSTAR Max DNA Polymerase (Takara Bio Inc., Otsu, Japan) using the following primer pair: 5'-TCG TGA AGT GCC ACC ATG TCC CAT CCT CAG GAG CCG GAG-3' (forward) and 5'-CTC CTC GCC CTT GCT CAC GAG GAA GGA ATC CAC AGG AGG-3' (reverse) and a mouse brain cDNA library (Clontech, Mountain View, CA, USA) as a template. Amplified fragments were cloned into the pENTR/D-TOPO vector using the In-Fusion Advantage PCR Cloning Kit (Takara Bio Inc.) according to the manufacturer’s instructions. For the constitutively active SGK1

construct, SGK1-S422D was amplified from pENTR-SGK1-GFP using the following primer set: 5'-GCC TTC CTC GGC TTC GAC TAT GCA CCT CCT GTG-3' (forward) and 5'-TTC TGC TGC TTC CTT CAC ACT GGC-3' (reverse) (the mutated bases shown as lowercase letters cause a Ser to Asp substitution at amino acid 422). Recombinant adenoviruses expressing GFP alone or fused to mouse SGK1 or N-myc downstream regulated (NDRG)1 were generated using the ViraPower Adenoviral Expression system (Life Technologies, Inc.) according to the manufacturer's instructions.

Cell culture. Primary oligodendroglial cultures were prepared from P1 Wistar rat cortex using a previously described method[4]. Briefly, the cortex was placed in a poly-L-lysine-coated flask in α-Minimal Essential Medium containing 10% heat-inactivated fetal bovine serum at 37°C in an atmosphere of 95% air/5% CO_2 for 14 days with shaking at 200 rpm. Cells were then spread in poly-L-lysine-coated flasks at a density of 1×10^4 cells/cm^2 in Neurobasal Medium (Life Technologies, Carlsbad, CA, USA) containing B27 supplement, platelet-derived growth factor (PDGF), neurotrophin

3, and insulin and incubated for 3 days, after which the medium was replaced with PDGF-free Neurobasal Medium. Cells were transfected using Lipofectamine LTX and Plus regent or Lipofectamine RNAiMAX (Life Technologies) according to the manufacturer's instructions.

Primary OL–dorsal root ganglion neuron co-cultures. Primary rat OLs and mice DRG neuron co-cultures were prepared using P1 Wister rat cortex and C57/BL6 mice DRG neurons with a previously described method[5–7]. Briefly, DRG neurons were obtained from E13.5 mice embryos and dissociated by pipetting in 0.25% trypsin and 0.4% DNase at 37°C for 30 min until a single-cell suspension was obtained. Cells were then seeded in poly-L-lysine-coated 4-well Lab-Tek Chamber Slide at a density of 4×10^4 cells/well in Neurobasal Medium (Life Technologies) containing B27 supplement, D-glucose, L-glutamine, nerve growth factor, and 5 mM fluorodeoxyuridine for 14–21 days. The medium was replaced with a DRG-OL co-culture medium (1:1 Dulbecco's Modified Eagle's Medium/F12 + Neurobasal medium with 1% N2 supplement, 2% B27 supplement, 2 mM glutamine, 10 ng/ml biotin, and 30 ng/ml T3) and 0.5×10^4 OPC

cells were added to each well. After 14 days the cells were fixed with 4% paraformaldehyde for analysis.

Short interfering (si)RNA knockdown. Stealth siRNA against *Sgk1* (5'-GAA GCA UUC UAU GCC GUC AAA GUU U-3') and negative control duplexes (i.e., scrambled siRNA against *Sgk1*, 5'-GAA CUU AGU AUU GCC AAA CGC GUU U-3') were purchased from Life Technologies Inc.

Reverse transcription and RT-PCR. Total RNA was prepared from the corpus callosum of stressed and control mice using ISOGEN (NipponGene, Toyama, Japan) according to the manufacturer's instructions. The RNA was reverse transcribed using oligo(dT)12–18 primers and SuperScript III RNaseH reverse transcriptase (Life Technologies, Inc.) according to the manufacturer's instructions. RT-PCR was performed on an ABI PRISM 7900HT Sequence Detection System using SYBRGreen PCR Master Mix (Applied Biosystems, Foster City, CA, USA) according to the manufacturer's instructions. *Sgk1* and *mGluR1-5* expression levels were quantified

using the following forward and reverse primers: *Sgk1*, 5'-GGG TGC CAA GGA TGA CTT TA-3' (complement of bases 1020–1039) and 5'-CTC GGT AAA CTC GGG ATC AA-3' (reverse complement of bases 1154–1173); *mGluR1*, 5'-CTA AGA AGC CTA TTG CTG GA-3' (complement of bases 455–474) and 5'-AGT TTT GTC GCT CAG GTC TA-3' (reverse complement of bases 569–588); *mGluR2*, 5'-CTT CCC ATC TTC TAC GTC AC-3' (complement of bases 2329–2348) and 5'-AGA GGA TAA TGT GCA GCT TG-3' (reverse complement of bases 2436–2455); *mGluR3*, 5'-TGT GGC TTA TCT TGG AGA CT-3' (complement of bases 2114–2133) and 5'-AAC CAC GTC ATA GGT CAG AG-3' (reverse complement of bases 2219–2238); *mGluR4*, 5'-CAT GTC CAA CAA GTT CAC AC-3' (complement of bases 2604–2623) and 5'-AGT GAC GTA GGT CTG TTT GG-3' (reverse complement of bases 2699–2718); and *mGluR5*, 5'-GCA GGG GAA TTT CTA CTT CTG G-3' (complement of bases 883–905) and 5'-ACC ACT TGA CAT CAG GAG AC-3' (reverse complement of bases 987–1006). GAPDH served as an internal control and was amplified using the following primers: 5'-GTG TTC CTA CCC CCA ATG TG-3' (forward) and 5'-AGG AGA CAA CCT GGT CCT CA-3' (reverse).

Immunocytochemistry. Primary oligodendrocytes were cultured on four-well chamber dishes at a density of 3 × 10^4 cells/cm^2, then fixed in 4% paraformaldehyde in 0.1 M PBS and incubated at 4°C in a humid atmosphere for 1 day with an anti-GFP antibody diluted 1:200 in PBS containing 0.3% Triton X-100 and 5% bovine serum albumin. The cells were then rinsed with PBS for 1 h and incubated at room temperature for 2 h with Alexa Fluor 488-conjugated goat anti-rabbit IgG or Alexa Fluor 568-conjugated goat anti-mouse IgG (Life Technologies, Inc.) diluted 1:500 in PBS. The cells were washed with PBS for 1 h and mounted with PermaFluor (Thermo Scientific, Waltham, MA, USA), and visualized with an LSM-510 confocal microscope (Carl Zeiss, Oberkochen, Germany) under 20× objective. Morphometric measurements were made using ImageJ software (National Institutes of Health, Bethesda, MD, USA).

Na^+/K^+-ATPase activity assay. Na^+/K^+ ATPase levels in the corpus callosum were measured using a Mouse ATPase (ATPase, Na^+/K^+ Transporting) enzyme-linked immunosorbent assay kit (Elabscience, Hubei, China) according to the manufacturer's

protocol. ATPase activity levels in the corpus callosum were determined using a QuantiChrom ATPase/GTPase Assay kit (BioAssay Systems, Hayward, CA, USA) according to the manufacturer's protocol but with modifications. Briefly, homogenates of the corpus callosum were prepared in TNE buffer (50 mM Tris–HCl, pH 7.4; 100 mM NaCl; and 0.1 mm EDTA) and freeze-thawed four times. Reactions were prepared by combining 20 μl assay buffer, 10 μl lysate, and 10 μl of 4 mM ATP. A 200-μl volume of Malachite Green reagent was added to each reaction, followed by incubation for 30 min at room temperature. Optical density was measured at 620 nm on a plate reader. Protein amounts were normalized to the total protein content of each sample prior to quantitative analysis.

Membrane potential assay. Primary neurons, OPCs, and mature OLs were cultured in Greiner high-binding 96-well plates (black wells with clear flat bottoms) in 50 μl Neurobasal Medium containing B27 supplement. An equivalent volume of loading buffer from the FLIPR Membrane Potential Assay kit (Molecular Devices, Sunnyvale, CA, USA) was added to each well, and the plates were incubated for 30 min at 37°C in

an atmosphere of 95% air/5% CO_2; membrane potential levels were determined using the kit according to the manufacturer's protocol[8]. Absorbance was measured using a Varioskan Lux multimode Microplate reader (Thermo Fisher Scientific, Waltham, MA, USA).

Measurement of cellular cAMP levels. Levels of cAMP in the corpus callosum were determined using the cAMP-Glo Assay kit (Promega, Madison, WI, USA) according to the manufacturer's protocol but with modifications. Briefly, corpus callosum homogenates were prepared in TNE buffer and freeze-thawed four times. Reactions were carried out by adding 20 µl cAMP-Glo lysis buffer and incubating for 15 min at room temperature; 40 µl cAMP-Glo detection solution were then added, followed by a 20-min incubation at room temperature. Finally, 80 µl Kinase-Glo reagent were added and the sample was incubated for 10 min at room temperature. A Wallac ARVO MX 1420 Multilabel Counter (Perkin Elmer, Waltham, MA, USA) was used to measure luminescence. Protein amount was normalized to the total protein content of each sample prior to quantitative analysis.

DTI study. We recruited participants with MDD from the outpatient clinics of the Department of Psychiatry, Osaka University Medical School. The diagnoses of MDD in all patients were made independently by 2 trained psychiatrists (T.K. and F.Y.) using the Structured Clinical Interview for DSM-IV Axis I Disorders (SCID)[9]. Patients were required to fulfill DSM-IV criteria for MDD, have no comorbid Axis I diagnosis, have a score of at least 14 on the 17-item Hamilton Rating Scale for Depression (HAM-D)[10] and have no use of psychotropic medication, electroconvulsive therapy or psychotherapy at the time of examination. We recruited participants for the healthy control group from the community. The control participants were required to have no personal history of a DSM-IV Axis I disorder; this was confirmed using the SCID[9]. The exclusion criteria for all participants were any contraindications for MRI, a history of head trauma with loss of consciousness for 5 or more minutes or any neurological disorder and any concomitant major medical disorder. The MDD group included 12 participants (mean age 38.4 [standard deviation (SD) 7.1] year, range 27–49 year, 67% women, mean HAM-D score 23.8 [SD 6.6]). The control group included 14 participants

(mean age 34.2 [SD 5.3] year, range 28–45 year, 36% women, mean HAM-D score 1.6 [SD 2.1]). All participants were right-handed. The MDD and control groups did not differ significantly in age or sex (all $P > 0.05$).

All MRI examinations were performed with a 3T whole-body scanner (Signa Excite HD V12M4; GE Healthcare, Milwaukee, WI, USA) with an 8-channel phased-array brain coil. DT images were acquired with a locally modified single-shot echo-planar imaging (EPI) sequence by using parallel acquisition at a reduction (ASSET) factor of 2 in the axial plane. Imaging parameters were as follows: TR = 17 s; TE = 72 ms; b = 0, 1000 s/mm^2; acquisition matrix, 128 × 128; field of view (FOV), 256 mm; section thickness, 2.0 mm; no intersection gap; 74 sections. The reconstruction matrix was the same as the acquisition matrix, and 2 × 2 × 2 mm isotropic voxel data were obtained. Motion probing gradient (MPG) was applied in 55 directions, the number of images was 4,144, and the acquisition time was 15 min 52 s. To reduce blurring and signal loss arising from field inhomogeneity, an automated high-order shimming method based on spiral acquisitions was used before acquiring DTI scans[11]. To correct for motion and distortion from eddy current and B0 inhomogeneity, FMRIB

software (FMRIB Center, Department of Clinical Neurology, University of Oxford, Oxford, England; http://www.fmrib.ox.ac.uk/fsl/) was used. B0 field mapping data were also acquired with the echo time shift (of 2.237 ms) method based on two gradient echo sequences.

Imaging processing and analysis. FA maps and 3 eigenvalues (l_1, l_2, and l_3) were generated from each individual using FMRIB software. First, brain tissue was extracted using the Brain Extraction Tool in FSL software. Brain maps for each of the 55 directions were eddy-corrected, and then FA values were calculated at each voxel using the FSL FMRIB Diffusion Toolbox. Image preprocessing and statistical analysis were carried out using SPM8 software (Wellcome Department of Imaging Neuroscience, London, England). Each subject's echo planar image was spatially normalized to the Montreal Neurological Institute echo planar image template using parameters determined from the normalization of the image with a b value of 0 s/mm^2 and the echo planar image template in SPM8. Images were resampled with a final voxel size of 2 × 2 × 2 mm^3. Normalized maps were spatially smoothed using an isotropic Gaussian filter

(8 mm full width at half maximum).

Voxel-based analysis was performed using SPM8 software. FA maps were compared between patients and healthy subjects using analysis of covariance (ANCOVA) with age and gender as covariates of no interest. We included age and gender as covariates because it was reported that they affect the white matter integrity[12]. Statistical inference was done with a voxel-level threshold of $P < 0.001$, uncorrected with a minimum cluster size of 50 voxels. The regional FA value in the anterior genu of the corpus callosum was calculated by averaging the FA values for all voxels within the spherical VOIs (3 mm radius) placed on the regions (x, y, z = –8, 32, 10 and 8, 32, 10 with MNI coordinates) of normalized individual FA maps. The same VOIs were applied to l_1—l_3 images, and l_1—l_3 values were extracted. Axial (l_1) and radial diffusivity ($[l_2+l_3]/2$) were compared.

Statistical analysis. Differences in demographic characteristics between patients and healthy controls were assessed with the unpaired t and Pearson's χ^2 tests. To examine between-group differences in FA values and axial/radial diffusivity in voxels of interest

(VOIs) in the voxel-based analysis, we performed an analysis of covariance with age and gender as covariates. All statistical tests were two-tailed and are reported at $\alpha < 0.05$. Bonferroni correction was applied to avoid type I errors due to the multiplicity of statistical analyses. Data were analyzed using SPSS for Windows 19.0 (IBM Japan, Tokyo, Japan).

Supplemental References

1. Rajasekharan, S. *et al*. Netrin 1 and Dcc regulate oligodendrocyte process branching and membrane extension via Fyn and RhoA. *Development*. **136**, 415-426 (2009).

2. Miyata, S. *et al*. Plasma corticosterone activates SGK1 and induces morphological changes in oligodendrocytes in corpus callosum. *PLoS One* **6**, e19859 (2011).

3. Miyata, S., Mizuno, T., Koyama, Y., Katayama, T. & Tohyama, M. The endoplasmic reticulum-resident chaperone heat shock protein 47 protects the Golgi apparatus from the effects of O-glycosylation inhibition. *PLoS One* **8**, e69732 (2013).

4. Chen, Y. *et al*. Isolation and culture of rat and mouse oligodendrocyte precursor cells. *Nat. Protoc.* **2**, 1044-1051 (2007).

5. Wang, Z., Colognato, H. & Ffrench-Constant, C. Contrasting effects of mitogenic growth factors on myelination in neuron-oligodendrocyte co-cultures. *Glia*. **55**, 537-545 (2007).

6. Laursen, L.S., Chan, C.W. & Ffrench-Constant, C. An integrin-contactin complex regulates CNS myelination by differential Fyn phosphorylation. *J. Neurosci*. **29**, 9174-9185 (2009).

7. Laursen, L.S., Chan, C.W. & Ffrench-Constant, C. Translation of myelin basic protein mRNA in oligodendrocytes is regulated by integrin activation and hnRNP-K. *J Cell Biol*. **192**, 797-811 (2011).

8. Baxter, D.F. *et al.* A novel membrane potential-sensitive fluorescent dye improves cell-based assays for ion channels. *J. Biomol. Screen*. 7, 79-85 (2002).

9. First, M.B., Spitzer, R.L., Gibbon, M. & Williams, J.B.W. Structured Clinical Interview for DSM-IV (SCID-I)—Research Version Biometrics Research, New York (1997).

10. Hamilton, M. A rating scale for depression. *J. Neurol., Neurosurgery & Psychiatry* **23**, 56–62 (1960).

11. Kim, D.H., Adalsteinsson, E., Glover, G.H. & Spielman, D.M. Regularized higher-order in vivo shimming. *Magn. Reson. Med.* **48**, 715-722 (2002).

12. Inano, S., Takao, H., Hayashi, N., Abe, O. & Ohtomo, K. Effects of age and gender on white matter integrity. *AJNR. Am. J. Neuroradiol.* **32**, 2103-2109 (2011).

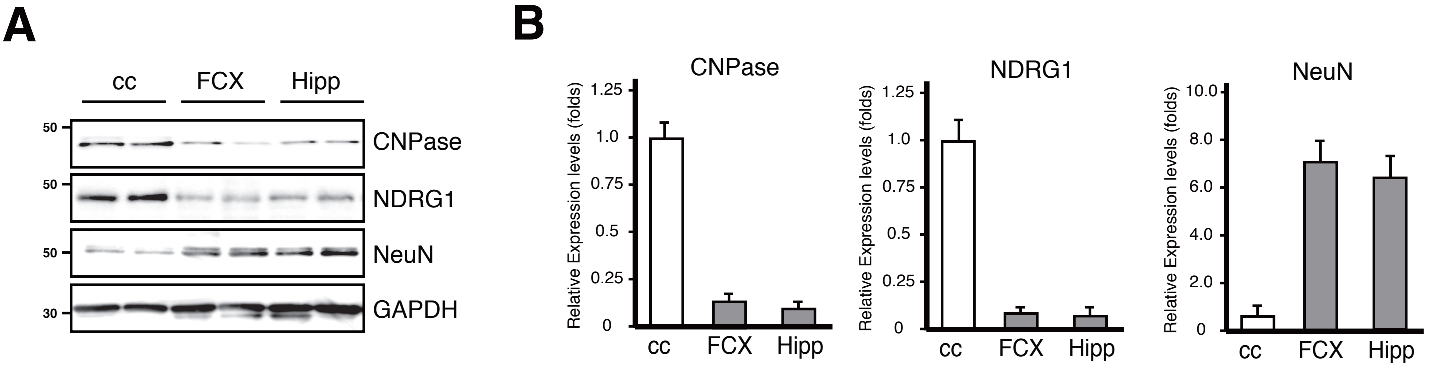

Supplementary Figure S1

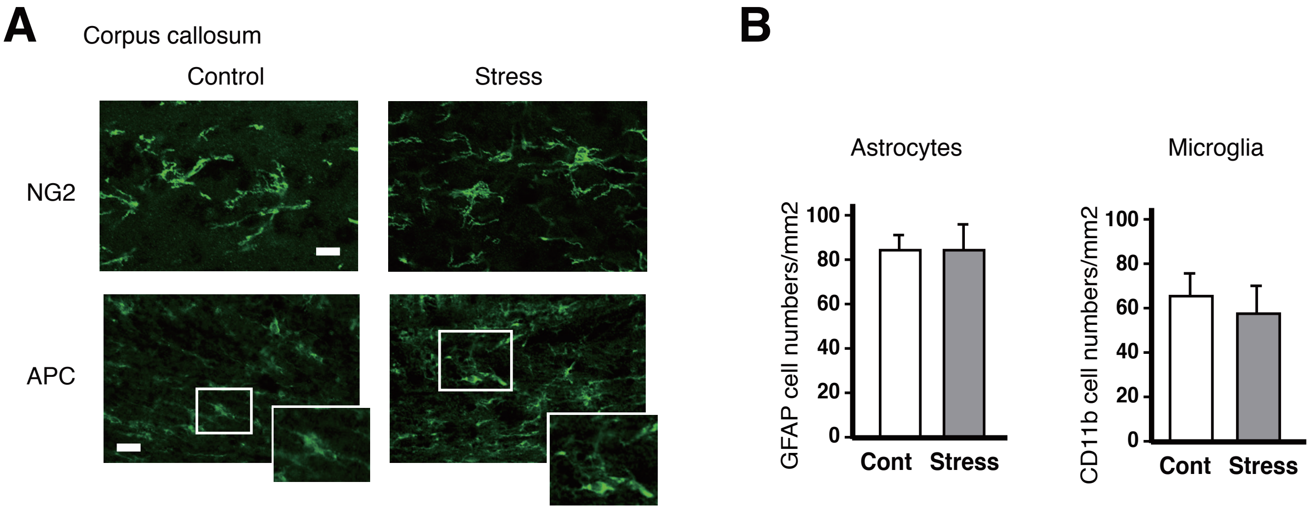

Supplementary Figure S2
